# Effect of pedo-climatic conditions on physicochemical characteristics and agro-industrial potential of three native oilseeds fruits from Burkina Faso

**DOI:** 10.1186/s12870-022-03713-7

**Published:** 2022-07-04

**Authors:** Hemayoro Sama, Dieudonné Kimbié Traoré, Samson Guenné, Adama Hilou, Mamoudou H. Dicko

**Affiliations:** 1 Laboratory of Biochemistry and Chemistry Applied (LABIOCA), University Joseph Ki-Zerbo, 03 Box 7021, Ouagadougou 03, Burkina Faso; 2 Laboratory of Biochemistry, Biotechnology, Food Technology and Nutrition, (LABIOTAN), Department of Biochemistry and Microbiology, University Joseph KI-ZERBO, BP 848 Ouagadougou 09, Burkina Faso

**Keywords:** Pedoclimatic, Seeds traits, Oilseeds, Agro-industrial potential

## Abstract

**Background:**

Plants are greatly affected by pedoclimatic conditions. They can alter the physiology of plants and affect seeds agro-morphological and physicochemical characteristics. It is therefore conceivable that tree species which have a potential as oil/fat producing plants are affected by pedoclimatic conditions variability. This study aims to evaluate the effect of pedoclimatic conditions variation on the physicochemical characteristics and the agro-industrial potential of three oilseeds fruits  from Burkina Faso: *Balanites aegyptiaca, Sclerocarya birrea and Lannea microcarpa.*

**Results:**

A characterization of the size, chemical composition and weight of 100 seeds of the three native oilseeds from Banfora (Sudanian zone), Ouagadougou (Sudano-Sahelian zone) and Ouahigouya (Sahelian zone) was carried out. Results showed that seed size, seed weight and chemical composition varied significantly according to the pedoclimatic zone of the collection  significant correlations between seed size, 100-seed weight, total ash and also for seed oil content and moisture have also been revealed. Principal component analysis (PCA) associated increases in seed size and total ash content with high annual rainfall and low temperature areas, while increases in seed oil content were associated with low rainfall and high annual temperature areas.

**Conclusion:**

Seed size and seed weight were associated with high rainfall and low temperature, while high temperature and low rainfall were associated with oil accumulation in the seeds. However, the limit number of replications of physicochemical characteristics analyses, a limitation of the study, does not allow an exhaustive conclusion to be drawn from the study.

## Background

Environmental conditions especially pedoclimatic conditions a have a remarkable effect on the life of living organisms, in particular species growth and distribution [1, 2]. Pedoclimatic conditions can affect plants distribution, production and physiology and can also affect seed characteristics (size, weight, etc.) and chemical composition [3, 4]. Indeed, stress (biotic or abiotic) negatively affects seed oil content and quality and significantly reduces their economic values. Soil properties by influencing water and nutrient uptake has a great effect on plant growth and seeds characteristics. Also, water availability was reported to have significant effect on plants productivity, seeds yield and seeds morphology [1].

In many African countries, demand for non-timber forest products is increasing and oil and fat producing plants in particular have great potential [5]. Natural oil sources usually contain stabilizing compounds, which can be used, supplemented with various antioxidants to further extend storage or use lifetime [5]. The need to find new sources of oilseeds and natural preservatives of oils has led to initiate researches into the native flora, especially the African one. Previous ethnobotanical investigations on oilseeds from Burkina Faso had revealed that fruits from trees such as *Balanites aegyptiaca* (L) Del. (soapberry three), African grappe *(Lannea microcarpa)* and Marula *(Sclerocarya birrea*) (A. Rich.) Hochst, possess high potentials [6]. The oils from the oilseed of these three tree species contribute enormously to the needs of rural population and are frequently used for food, cosmetics and traditional medicine by local people [5]. *Lannea microcarpa* plant organs (leaves, bark, fruits and gum) are traditionally used against gastroenteritis, female infertility, high blood pressure, edema, coughing, poisoning, and burn [5, 7] and in the manufacture of clothing [8]. *B. aegyptiaca* seed kernels are considered as an extremely useful edible product. It contains good quality oil and high protein content [9, 10]. The debittered kernels are used as snacks (nuts) by humans. The extracted oil used for many uses and the remaining cake is used as fodder [9]. Both fruits and kernels were widely used in many countries during the dry season and drought periods. *S. birrea* organs (flesh with peel) are used to treat diabetes, gastroenteritis, high blood pressure, and scurvy [5, 6]. Beside nutritional and bio-protection (anti-diabetic, anti-hypertensive, antioxidant, etc.) properties known of wild plants, wild oil seeds usually contain stabilizing compounds, which can be supplemented with various antioxidants to further extend storage or use lifetime of theirs oils [5].

In Burkina Faso, these species are widely dispersed in different climatic zones with various pedoclimatic conditions. It can be expected that they are detrimental to the human economy, especially as previous work has shown that some species can be negatively affected by drought, rain full, soil and temperature [11, 12]. This study was initiated in order to assess the effect of pedoclimate variability on the size, weight, chemical composition and agro-industrial potential of local oilseeds in order to identify the climatic zone that optimizes the economic value of each species.

## Results

### Variation in seed size and seed weight in relation to pedoclimatic zone of origin

#### Morphometric characteristics of seeds

**Fig. 1:**
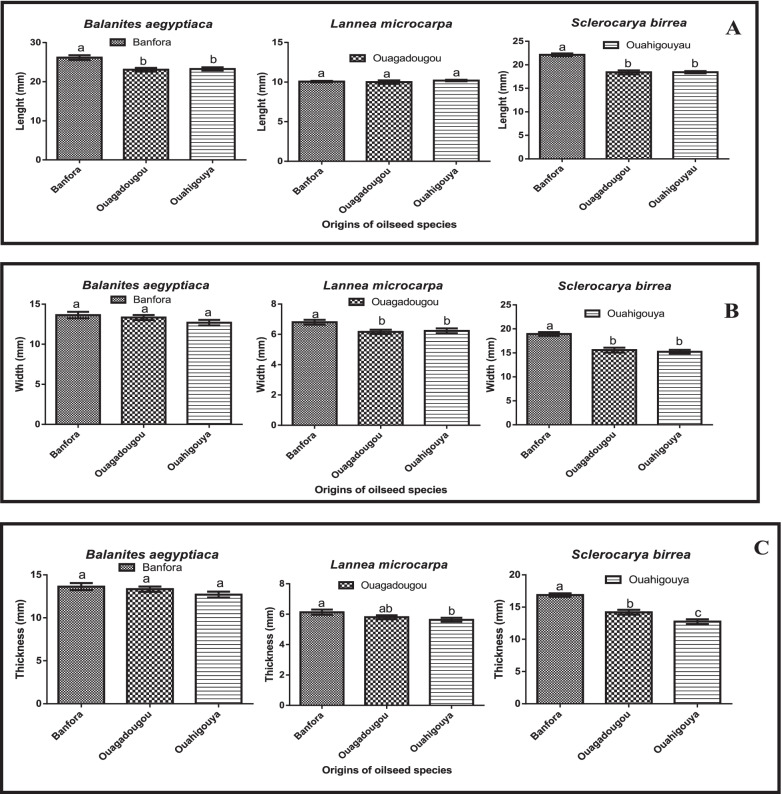
Variation in seed morphometric traits according to their origin of the 3 local plants. **A** variation in seed length of species according to origin; **B** variation in seed width of species according to origin; **C** variation in seed thickness of species according to origin. Histograms with different letters are statistically different at the 5% level

Data of the measurements of the seed size of the three oilseed species of the three climatic zones of Burkina Faso (Fig. 1) showed, in general, a significant variation (*p* < 0.001) in seed size depending on the species and origin for some morphometric traits. *L. microcarpa* seeds showed the lowest value of seeds size compared to the other two species. The highest seed length value (26.17 ± 3.24 mm) of *B. aegyptiaca*, was recorded in Banfora (southern Sudanian zone) while the lowest value (23.27 ± 2.36 mm) was from Ouahigouya (Sahelian zone). For *L. microcarpa* significant variations were also recorded in seed length. The highest value of width (6.80 ± 0.84 mm) and thickness (6.13 ± 0.93 mm) were recorded in Banfora (southern Sudan Zone). *S. birrea* showed significant variation in all morphometric traits. Thus, the highest value of length (22.13 ± 1.56 mm) was recorded in Banfora (southern Sudanian zone) while the lowest value (18.43 ± 1.38 mm) was recorded in Ouahigouya (Sahelian zone). The high value of width (18.93 ± 2.14 mm) and thickness (16.87 ± 1.45 mm) were recorded in Banfora (southern Sudanian zone).

#### Weight of 100 seeds

**Fig. 2:**
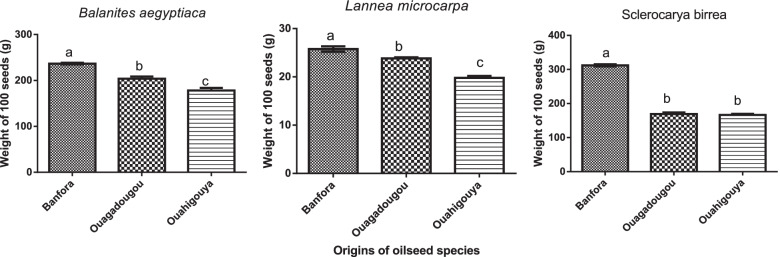
Variation in weight of 100 seeds in relation to species and origin. Histograms with different letters are statistically different at the 5% level

The results show a significant variation in the weight of 100 seeds depending on the species and the collection area (Fig. 2). In general, seeds from Banfora (southern Sudanian zone) presented the highest weight while those from Ouahigouya (Sahelian zone) had the lowest weight. In *B. aegyptiaca*, the highest value weight of 100 seeds was 236.735 ± 3.11 g while the lowest was 178.14 ± 9.95 g. In *L. microcarpa* and *S. birrea*, the highest value of weight of 100 seeds was 25.77 ± 0.94 g and 312.11 ± 5.55 g respectively while the lowest was 19.80 ± 0.67 g and 166.451 ± 5.60 g.

#### Physicochemical characteristics of seeds according to their origin

Data on physicochemical characteristics showed a significant variation of certain parameters such as moisture, total starch and seeds oil contents and also according to the species and on the other hand according to the zone (Table 1). The residual moisture varied significantly only in *B. aegyptiaca*. The highest value of residual moisture content was recorded in Ouagadougou (4.65 ± 0.22%) while the lowest was recorded in Banfora (4.24 ± 0.01%). For the other species, non-significant variations were recorded between provenances. For total ash, *S. birrea* seeds displayed the highest value compared to the other species. In addition, in *B. aegyptiaca* and *S. birrea*, the highest value was 3.11 ± 0.10% and 4.20 ± 0.26%, recorded in Ouagadougou (Sudanian zone) and Banfora (southern sudanian zone), respectively. The lowest ash content (2.46 ± 0.03% and 3.42 ± 0.08%) were recorded in Ouahigouya (sahelian zone). *L. macrocarpa* from Ouahigouya recorded the highest total ash content (3.05 ± 0.07%) while the lowest value (2.80 ± 0.05%) was recorded in Ouagadougou.

**Table 1:** Chemical composition of the seeds depending on geographic origin

Species	Origins	Moisture (%)	Total ash (%)	Oil content (%)
***Balanites aegyptiaca***	Banfora	4.24 ± 0.01^b^	2.84 ± 0,11^b^	42.18 ± 1.10^c^
	Ouagadougou	4.65 ± 0.22^a^	3.10 ± 0,10^a^	47.03 ± 0.97^a^
	Ouahigouya	4.41 ± 0.06^ab^	2.45 ± 0,03^c^	44.51 ± 0.13^b^
***Lannea microcarpa***	Banfora	4.64 ± 0.10^a^	2.85 ± 0,08^b^	59.47 ± 0.63^a^
	Ouagadougou	4.62 ± 0.30^a^	2.80 ± 0,05^b^	57.32 ± 0.13^b^
	Ouahigouya	4.63 ± 0.32^a^	3.04 ± 0,07^a^	55.36 ± 0.67^c^
***Sclerocarya birrea***	Banfora	4.10 ± 0.08^a^	4.19 ± 0.26^a^	51.30 ± 9.93^a^
	Ouagadougou	4.35 ± 0.13^a^	3.57 ± 0.04^b^	51.81 ± 8.05^a^
	Ouahigouya	4.17 ± 0.14^a^	3.41 ± 0.08^b^	56.23 ± 0.57^a^

For each species, the columns with different letters (a, b and c)are statistically different at the 5% level

The oil content of the seeds also varied significantly according to species and also according to the collection area. Thus, *B. aegyptiaca* generally showed relatively low oil contents compared to the other two species. The highest oil contents (47.03 ± 0.97% and 59.47 ± 0.63% % of seeds powder) were recorded in Ouagadougou and Banfora for *S. birrea* and *L. macrocarpa,* respectively. The lowest values (42.18 ± 1.10% and 55.36 ± 0.67%) were recorded in Banfora and Ouahigouya respectively for the same species. Non-significant variations in oil content were recorded for *S. birrea* seeds.

#### Correlations between studied parameters and PCA analysis-based seed characteristics

Pearson linear correlation coefficients revealed positive and significant correlations between seed size, 100-seed weight and total ash and also between seeds oil content and moisture (Table 2). The length and width of the seeds were correlated with the weight of the 100 seeds with a coefficient of 0.98. The oil content was negatively related to the length (*R* = -0.99), width (*R* = -0.98) and weight of 100 seeds (*R* = 0.99).

**Table 2:** Pearson correlation matrix of evaluated parameters^a^

Variables	Weight of 100 Seeds	Total ash	Moisture	Oil content	Thickness	Width	Length
Weight of 100 Seeds	**1**						
Total ash	0.89	**1**					
Moisture	-0.68	-0.27	**1**				
Oil content	**-0.99**	-0.81	**-0.99**	**1**			
Thickness	**0.96**	**0.98**	**0.96**	**0.96**	**1**		
Width	**0.99**	0.90	**0.99**	**0.99**	**0.99**	**1**	
Length	**0.98**	0.78	**0.98**	**0.98**	**0.98**	**0.98**	**1**

^a^Values in bold are different from 0 at significance level alpha = 0.05

**Fig. 3:**
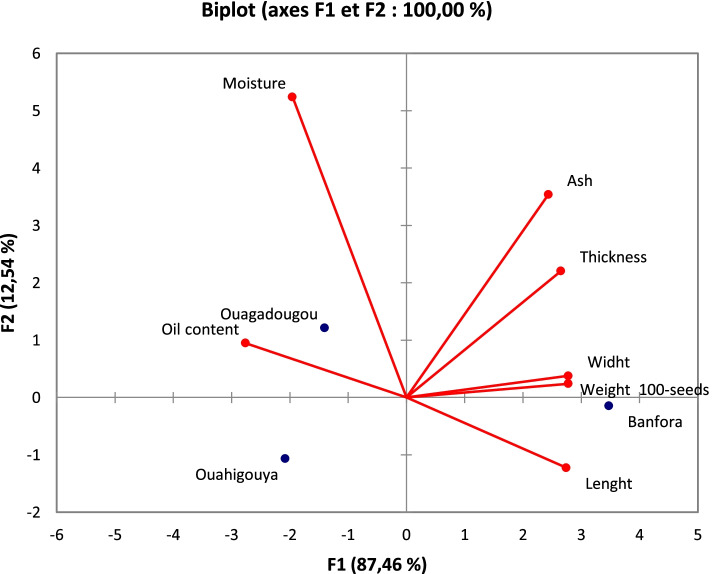
PCA based on seed traits. ACP shows the distribution of seed collection sites with their associated seed traits

Principal component analysis (PCA) was carried out with the objective of associating, where possible, the size, 100-seed weight and chemical composition of the seeds of the three species with climatic conditions. The selected axes explain 100% of the total variation with 87.46% and 12.54% for axes 1 and 2 respectively. Figure 3 illustrates a distribution of the different collection sites according to a factorial design with their associated seed characteristics. The axis divides the different provenances into two groups. Group 1 is the provenance of Banfora (sudanian zone), which is characterized by the seeds with the high size, the high weight, and the high ash content. Group 2, which includes provenances of Ouagadougou and Ouahigouya, is characterized by the seeds with the high oil and moisture contents. These results suggest that areas with high rainfall and moderate annual temperature, as is the case in the Banfora area, were favorable for obtaining large seeds with a high 100-seed weight. As for areas with high temperatures and low or moderate rainfall, such as Ouagadougou and Ouahigouya, they were favorable for obtaining seeds with high oil content.

## Discussion

Nowadays, the need for non-timber forest products at local and global level is increasing [5]. However, plants are greatly affected by pedoclimatic conditions [1]. Indeed, pedoclimatic conditions can alter the distribution, production and physiology of plants and can affect seed characteristics (size, weight, etc.) and chemical composition [4, 13]. Burkina Faso, like many regions of the world and Africa, is characterized by a great pedoclimatic diversity. This high pedoclimatic diversity could harm the human economy by affecting some local species with high socio-economic potential. *Balanites aegyptiaca, Sclerocarya birrea* and *Lannea microcarpa* are three local oleaginous tree species widely distributed in all geographical areas of Burkina Faso with known high socio-economic potential. In order to identify the climatic zone that optimizes the economic value of the local oil/fat producing plants this study was initiated.

The study revealed a variation in seeds size, weight, and chemical composition depending to origin for all the three species. These results suggested that seed traits may be strongly affected by environmental variations. Indeed, as the populations of the three species in the three study areas are natural populations, this variability could be explained by environmental conditions [14]. Results also show that as one moves from the south of the country, a zone with relatively high rainfall and moderated annual temperatures, to the north of the country (low rainfall and high annual temperature area), the seeds have a tendency to decrease in size and weight while the oil content increases. Relatively high rainfall seems to favor seed seeds size and weight. Similar results were reported by findings [14], which reported that seeds of carob trees, *Ceratonia siliqua* L. in the northern Morocco ecoregions, an area with high rainfall, were long, wide, heavy and thick. Research has also reported that the oil content of seeds is positively correlated to high temperatures [15]. Studying the effect of temperature on the variation of oil content in soybeans, [16] and [17] showed that the oil content increased with temperature up to an extreme value, beyond which the oil content decreased. This fact could explain the fact that seeds from Ouahigouya and Ouagadougou, areas with relatively high temperatures compared to the Banfora. Low rainfall and high temperatures lead to a reduction in seed size and an increase in seed oil content [4]. Similar results have been reported in plant species such as wheat [18] and maize [19]. This variability in seed traits depending to origin can be explained by an adaptive response to different ranges of climatic conditions prevailing in collection sites [20]. In order to overcome environmental conditions (temperature, rainfall, pollution, acidity, wind pressure, etc.), plant metabolisms may differ. These abiotic conditions can affect the characteristics of the seed, including its size, weight and biochemical composition [21]. Indeed, studies on other oilseeds such as rape have shown that when rainfall is very high, soil nitrogen was available and its uptake by the plant increased. The high amount of absorbed nitrogen could compete for carbon skeletons in the developing seed and possibly divert the available carbon to protein synthesis. When rainfall is low, soil nitrogen becomes unavailable and metabolism is diverted from seed metabolism to lipid synthesis and accumulation. This could justify the fact that areas with high rainfall have high seed size and weight; while those with low rainfall have small seed size and weight but high oil content. These results may also explain the positive and significant correlations recorded between seed size and weight and the negative ones recorded between seed oil content and seed size and weight. When rainfall and temperature conditions are favorable, protein synthesis is activated at the expense of lipid synthesis, resulting in large seeds with high weights and oil contents. When conditions become unfavorable, lipid accumulation takes over, resulting in small seeds that weigh less but have a high oil content.

Research has shown that climatic conditions of plant species including rainfall and temperature are the most important climatic parameters influencing species seeds size and seeds chemical composition [22]. High rainfall with moderate temperatures were associated with seeds of high size and weight while low rainfall with high temperatures were associated with seeds with high oil content and relatively low size. The results also showed significant positive correlations between seeds size and weight. Seed oil contents on the other hand, were negatively correlated with seeds size and weight. These results could be explained by the fact that when rainfall increased, seed length, width, thickness weight.

## Conclusion

Pedoclimatic conditions affect the daily life of living species and their physiology. Environmental conditions influencing the physiology of plant species can affect the characteristics of the seeds. This study was initiate in order to assess the effect of pedoclimate conditions on wild fruits. Results showed that high rainfall with moderate temperatures is associated with seeds with high size and weight while low rainfall with high temperatures is linked with seeds with high oil content and relatively low size. Data seem to suggest that areas with high annual temperatures and low rainfall are favorable for obtaining the small sized seeds with high oil content while areas with moderate temperatures and high rainfall are favorable for obtaining the large sized seeds with low oil content. However, the lack of replication of the physicochemical analysis in the different zones, an important limitation of the study, does not allow an exhaustive conclusion to be drawn from the study.

## Material and methods

### Presentation of the study areas

**Fig. 4:**
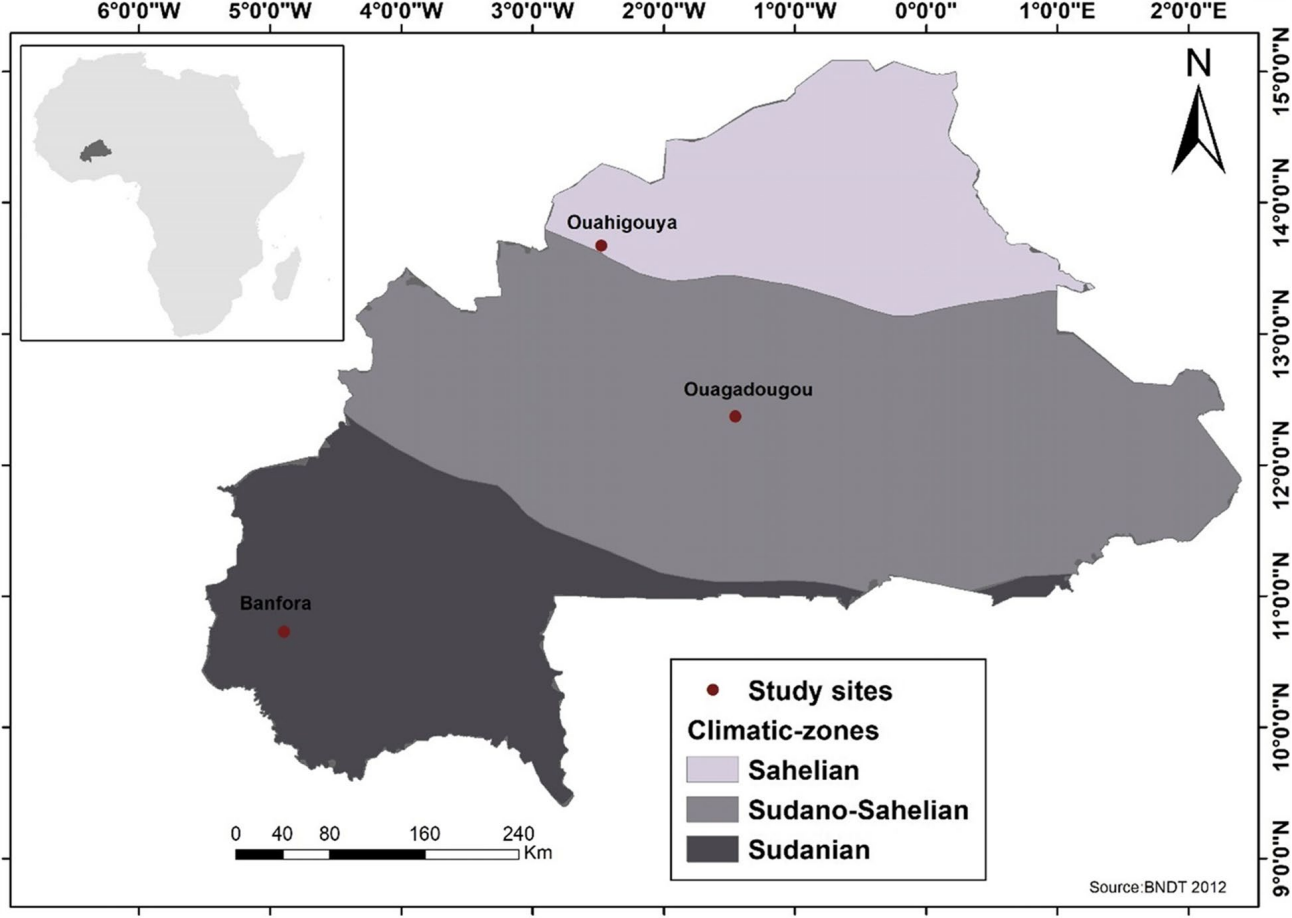
Location of the different collection sites

Samples were collected from surrounding villages in three communes (Fig. 4) of Burkina Faso: Banfora, Ouagadougou and Ouahigouya. These communes are located in the three main climatic zones of Burkina Faso: the Sudanian (Banfora), the Sudano-Sahelian (Ouagadougou) and the Sahelian (Ouahigouya).

### Sudanian zone

The Sudanian ecological zone (Banfora, 10° 37′ 60" North, 4° 46′ 0" West), mostly dominated by savanna communities. The climate is tropical with a unimodal rainy season, lasting for about 6 months from May to October. The mean annual rainfall is between 800 and 1200 mm, with 70–90 rainy days per year. The temperature in this area varies between 17 °C and 36 °C. This region has a relief made up of mountains, plateaus and plains with a soil that comes essentially from the alteration of old kaolinitic, quaternary deposits made up of eolian sands or alluvium. These soils are made up of a shallow, sandy-clay surface horizon, which becomes progressively clayey with depth, and often shows reddish concretions. reddish concretions. As for the vegetation, high rainfall and soils fertility allows for a very diversified vegetative formation which are: wooded savannah, tree savannah, open forest, gallery forest, and grassland [23].

### Sudano-sahelian zone

It is a region of climatic (Ouagadougou, 12° 21′ 56.4" North, 1° 32′ 2" West.) transition between the southern Sudanian zone and the Sahelian zone. It is a region characterized by a rainy season that extends from the beginning of June to the end of September with a rainfall of 700 to 900 mm per year. The climate is characterized by a wind that is very hot during the day, colder at night, very dry and usually dusty. and the monsoon. It is the largest climatic zone in the country. Its average temperature is around 28 °C. In the Sudano-Sahelian zone, the soils are predominantly tropical ferruginous and are characterized by a deficiency in organo-mineral colloids with low surface physicochemical activity. The vegetation is characterized by a very dense forest and a continuous herbaceous carpet.

### Sahelian zone

The Sahelian zone (Ouahigouya in our study, 13° 34′ 58" North, 2° 25′ 17.7" West.) represents almost 25% of the territory of Burkina Faso, with a rainy season that extends from June to September and a long dry season. The average annual rainfall is between 600 and 800 mm. It is area with the lowest rainfall of the country. It has an altitude that varies between 200 and 400 m and is characterized by the presence of plains and plateaus. It also has a very varied vegetation and soil. The geomorphology is characterized by dune strips of eolian origin which correspond to ancient and recent ergs. sub-arid brown soils and hydromorphic soils. Thus, the vegetation consists of adapted thorny shrubs.

The locality of Banfora presented the high values of annual rainfall and humidity while the lowest values are recorded in Ouahigouya (Table 3). The lowest annual temperature was recorded in Banfora while the highest was from Ouahigouya.

**Table 3:** Meteorological data for collection localities in 2020 [24]

Locality	Annual temperature (° C)	Annual rainfall (mm)	Humidity (%)
Banfora	30.58	1361	57
Ouagadougou	31.83	952	45
Ouahigouya	31.91	667	39

### Biological materials and sample collection

The plant material consisted of the ripe fruits of three oilseed of tree species (*Balanites aegyptiaca*, *Lannea microcarpa* and *Sclerocarya birrea*) from three pedoclimatic zones of Burkina Faso. In each zone, contact was made with local resource persons (village chief, landowners, etc.) to obtain their agreement to collect the fruits. The study was in accordance with the relevant national/institutional guidelines.

Fruits of the three species were collected in *Banfora* (Sudan zone), *Ouagadougou* (Sudano-sahelian and *Ouahigouya* (Sahelian). The fruit samples were collected between February and June 2020. The fruits of a natural population of the all three species in the three zones were collected in five sites (villages) per zone and per species, i.e. five replications per zone and per species. For each species and for each site, fruits from the five trees at the same stage of ripening, older than 5 years and at least 20 m apart were collected. The fruits from the five trees in each site per species were mixed and labelled differently. At this age, the productivity and seed characteristics of the oilseeds are considered stable. For each town, the fruits of the different plants are then mixed and constitute the sample. The collected fruits were cleaned of their pulp, then washed with running water and dried in the laboratory under ventilation. After drying, the seeds were packaged in plastic bags, stored at 4 °C and used for the various analyses.

### Morphological characterization of seeds

#### Morphometric characteristics

Seed size was determinate using a method described by [25]. For the morphometrical characterization of the seeds, measurements were carried out on 100 randomly selected seeds of each species and site. The morphological analyses were carried out on the five samples per zone, i.e. five replications per zone. The length, width and thickness of the seeds in each batch were then measured using an electronic caliper. The results were expressed as (Mean ± SD) mm.

#### Weight of 100 seeds

Weight of 100 seeds was determinate using an electronic precision balance [25]. The weight of 100 seeds was carried out on the five samples per zone, i.e. five replications per zone. The 100 seeds from each site and species were randomly selected and their weight measured. Weight averages per zone and species were calculated and the results expressed as (Mean ± SD) mg.

#### Chemical characterization of seeds

For the chemical analyses, samples from each zone were mixed to form a composite sample per zone. Each parameter was then analyzed in triplicate.

### Seed treatment

A batch of seed samples from each source was manually dehulled and the resulting kernels were ground using a blender. The resulting vegetable powder (paste) was used for the various analyses. This powder was used for the different analyses.

### Moisture content

Seeds moisture content was determined using French standard (03–603 V) as previously described [26]. A RADWAG moisture meter (MAC 110 series) equipped with a balance and a thermometer was used. The drying temperature was set at 60 °C according to WHO guidelines. Approximately 3 g sample of each plant powder was placed in the desiccator. After a few minutes (about 10 min), depending on the moisture level of the sample, the moisture content of the sample (in %) displayed on the screen was noted. The measurements were carried out in triplicate and averages were calculated and the results expressed as (Mean ± SD) %.

### Ash content

Seeds total ash content was determined according to the French standard (NF V 03–922) as described by [26]. Five grams of each sample were placed in an incinerator crucible with known empty mass. The crucible containing the sample was then introduced into the VOLCA type electric furnace set at 550 °C ± 15 °C. The furnace was kept at this temperature for at least 4 h until ash free of carbonaceous particles is obtained. The crucible was then placed in a desiccator and left to cool for 1 h and the crucible was weighed again. The analyses were carried out in triplicates and averages were calculated and the results expressed as (Mean ± SD) %. The total ash content is given by the formula:

$$\mathrm{Ash \ content }\left(\mathrm{\%}\right)=\frac{\left(Mt-M0\right)*100}{Sample \ weight}$$

M0: Empty mass of the crucible; Mt: Final mass of the crucible.

### Oil content

The seed oil content was determinate using the Soxhlet method as described by [25]. Oil in seeds was extracted for 6 h, using petroleum ether (boiling point of 40 -60 °C) as an extraction solvent. The extracted oil is recovered by solvent evaporation using a rotavapor apparatus to remove the majority of the solvent by rotatory evaporation at 40 °C under reduced pressure. The extracted seed oil was weighed. For each accession, the oil content of three samples was determined in triplicate tests in order to enhance accurate statistical inference. The amount of oil in seeds was calculated and expressed as percentage (%) by following formula:

$$\mathrm{Oil\ content }\ (\mathrm{\%}) =\frac{(Oil\ weight)*100}{Sample\ weight}$$

### Statistical analysis

The investigated parameters were subjected to one-way analysis of variance at the 5% level. For each specie, when the ANOVA test showed significant differences in seed traits between the different zones, the Tukey test (at the 5% threshold) was performed for the ranking of averages. The Pearson test was performed at the 5% threshold to assess the correlation between seed traits.

## Data Availability

The data that support the findings of this study are included in this published article.
